# Genomic actions of 1,25-dihydroxyvitamin D_3 _on insulin receptor gene expression, insulin receptor number and insulin activity in the kidney, liver and adipose tissue of streptozotocin-induced diabetic rats

**DOI:** 10.1186/1471-2199-9-65

**Published:** 2008-07-18

**Authors:** Consuelo Calle, Begoña Maestro, Moisés García-Arencibia

**Affiliations:** 1 Department of Biochemistry and Molecular Biology, School of Medicine, Complutense University, 28040-Madrid, Spain

## Abstract

**Background:**

this study set out to examine the effects of the treatment with 1,25-dihydroxyvitamin D_3 _(*1,25D*_3_) [150 IU/Kg (3.75 μg/Kg) one a day, for 15 days] to non-diabetic rats and in rats rendered diabetic by a single injection of streptozotocin [65 mg/kg].

**Results:**

treatment with *1,25D*_3 _to non-diabetic rats did not affect the biochemical parameters measured in the plasma and urine of these animals. Likewise, insulin receptor expression in the kidney, liver, or adipose tissue and insulin-stimulated glucose transport in adipocytes from these animals were not affected either. Treatment with *1,25D*_3 _to streptozotocin-induced diabetic rats did not correct the hyperglycemia, hypoinsulinemia, glycosuria or ketonemia induced by the diabetes, although it partially reversed the over-expression of the insulin receptor gene in the liver and adipose tissue, without altering the normal expression of this gene in the kidney. These effects were accompanied by a normalization of the number of insulin receptors without altering receptor affinity but improving the insulin response to glucose transport in adipocytes from these diabetic animals. Moreover, a computer search in the rat insulin receptor promoter revealed the existence of two candidate vitamin D response element (VDRE) sequences located at -256/-219 bp and -653/-620 bp, the first overlapped by three and the second by four AP-2-like sites.

**Conclusion:**

these genomic actions of *1,25D*_3 _could represent beneficial effects associated with the amelioration of diabetes via mechanisms that possibly involve direct transcriptional activation of the rat insulin receptor gene. The candidate VDREs identified may respond to *1,25D*_3 _via activation of the vitamin D receptor, although this remains to be investigated.

## Background

It is known that vitamin D and especially its activated metabolite 1,25-dihydroxyvitamin D_3 _(*1,25D*_3_), are involved in controlling the normal function of the endocrine pancreas, and particularly insulin secretion [1,2]. Vitamin D deficiency inhibits rat pancreatic secretion and turnover of insulin, leading to impaired glucose tolerance, while replacement therapy with *1,25D*_3_, is able to reverse these abnormalities [3,4]. *1,25D*_3 _also affects the insulin receptor (IR), the protein to which insulin must bind to carry out its multiple biological actions in the cells. In this respect, our group has reported the first demonstration that *1,25D*_3 _increased human IR mRNA levels, the IR number, and the insulin response in U-937 human promonocytic cells through mechanisms that involve direct transcriptional activation of the human IR gene [5-7].

In non-obese diabetic mice, vitamin D deficiency accelerates type 1 diabetes [8], while chronic administration of *1,25D*_3 _reduces the incidence of diabetes in these mice, principally by modulating immune mechanisms [9-11]. Streptozotocin-induced diabetes is another animal model of diabetes that comprises both toxic and inflammatory mechanisms [12]. Mononuclear infiltration and the altered morphology of islets coupled with the disappearance of beta cells are among the histological changes reported in the pancreas of these animals [12,13]. Indeed, hyperglycemia and hypoinsulinemia, have also been described in this experimental model [14,15]. This hypoinsulinemia was associated with increased insulin binding in the kidney [16,17] and liver [14,18] and with somewhat controversial insulin binding results in adipose tissue [15,19,20]. The increases in renal and hepatic IRs, were accompanied by elevated IR mRNA expression in both tissues, and it could be reversed by treatment with insulin [21-23]. Despite the different alterations in IR mRNA levels and insulin binding, streptozotocin-induced diabetic rats characteristically display insulin resistance [15,18,19]. In this diabetic model, the administration of 1,25D_3 _for 8 weeks was reported to improve diabetes attenuating pancreatic islet damage and decreasing the insulin requirements [12].

*1,25D*_3 _acts in its genomic effects as a ligand for the vitamin D receptor (VDR, NR1I1) [24]. This receptor is a member of the superfamily of nuclear receptors that regulates gene expression as a vitamin D-dependent transcription factor. It exerts this action by binding, preferentially as a heterodimer with the retinoid × receptor (RXR), to vitamin D response elements (VDREs) in the promoter regions of target genes [25]. A VDRE generally consists of two direct imperfect repeats of six nucleotides separated by a three-nucleotide spacer. The VDR occupies the 3' half-site, while the RXR binds to the 5' half-site [26]. Sequence variations have been detected in the 3' half-element, the 5' half-element, the spacer, and in the sequences flanking the VDREs and these variations appear to be important in receptor-binding efficiency [27,28]. The identification of VDREs has only been possible in a very limited number of vitamin D-regulated genes [26,27]. Our group has reported the first identification of a functional VDRE overlapped by a downstream AP-2-like site that specifically binds VDR in the human IR gene promoter [29]. This VDRE accounted for the transcriptional induction of this gene by *1,25D*_3 _in U-937 human cells [7,29].

With these antecedents, the aim of the present investigation was to study the effects of the treatment with *1,25D*_3 _[150 IU/Kg (3.75 μg/Kg) one a day, for 15 days] to non-diabetic and streptozotocin-induced diabetic rats. The results indicated that while treatment with *1,25D*_3 _had practically no effect on non-diabetic rats, the same treatment in streptozotocin-induced diabetic rats corrected in part the over-expression of the IR gene in liver and adipose tissue, although it did not revert the hyperglycemia, hypoinsulinemia, glycosuria or ketonemia of these diabetic animals. At the same time, it produced normalization of the IR number without alterations in the receptor affinity and with an improvement of the insulin response in terms of glucose transport in isolated adipocytes of these diabetic animals. In addition, a computer search in the rat insulin receptor gene promoter revealed the existence of two DNA sequences: -256/-219 bp and -653/-620 bp, the first overlapped by three and the second by four AP-2-like sites. These sequences represent two candidate VDREs that could respond to *1,25D*_3 _via activation of VDR, although this remains to be further investigated.

## Results

### Body weights and plasma values of glucose, insulin, 25-hidroxyvitamin D_3_, calcium, phosphorus and proteins

Treatment with *1,25D*_3 _to non-diabetic rats increased the rat body weights but did not affect any parameter in the plasma of these animals (Table 1). The injection of streptozotocin reduced body weights and induced hyperglycemia and hypoinsulinemia (Table 1). Treatment with *1,25D*_3 _to streptozotocin-induced diabetic rats increased body weights but did not revert the hyperglycemia and the hypoinsulinemia induced by the diabetes, while calcium and phosphorus plasma levels were increased and plasma levels of 25-hydroxyvitamin D_3 _were maintained in the normal range [30] (Table 1).

**Table 1:** Body weights and plasma values of the four groups of rats under study

	***Control***	***1,25D***_3_	***STZ***	***STZ + 1,25D***_3_
**Final weight (g)**	283 ± 1	290 ± 2^a^	242 ± 6^ab^	254 ± 3^abc^
**Glucose (mg/dl)**	125 ± 7	130 ± 2	420 ± 36^ab^	459 ± 16^ab^
**Insulin (ng/ml)**	1.6 ± 0.3	1.5 ± 0.2	0.7 ± 0.3^ab^	0.5 ± 0.1^ab^
**25-Hydroxy-vitamin D_3 _(ng/ml)**	19 ± 1	15 ± 1	15 ± 3	17 ± 3
**Calcium (mg/dl)**	7.5 ± 1	10 ± 1	10 ± 0.4	11 ± 1^a^
**Phosphorus (mg/dl)**	6.8 ± 1	8.7 ± 1	7.2 ± 0.5	11 ± 0.7^ac^
**Proteins (mg/dl)**	65 ± 1	66 ± 1	63 ± 0.9	64 ± 1

Sham-treated rats (*Control*), rats treated with *1,25D*_3 _[150 IU/Kg (3.75 μg/Kg) one a day, for 15 days] (*1,25D*_3_), streptozotocin-induced diabetic rats (*STZ*) and streptozotocin-induced diabetic rats treated with *1,25D*_3 _(*STZ+1,25D*_3_).

Values are the mean ± SEM of 6–12 determinations in each group.

^a ^p < 0.05 vs. *Control*-rats; ^b ^p < 0.05 vs. *1,25D*_3_-rats; ^c ^p < 0.05 vs. *STZ*-rats.

### Values of specific gravity, pH, leukocytes, nitrite, protein, glucose, ketones, urobilinogen, bilirubin and blood in urine

Treatment with *1,25D*_3 _to non-diabetic rats did not affect any of the above cited urine parameters under study. Streptozotocin injection induced glycosuria (> 500 mg/dl) and the appearance of leukocytes, ketones and blood in the urine of these animals. Treatment with *1,25D*_3_to streptozotocin-induced diabetic rats did not modify the glycosuria and the ketonuria of the diabetes, but partially decreased the number of leukocytes in urine (from 10–25 to 5–10 leukocytes/μl).

### Total DNA, RNA and protein content of the kidney, liver and epididymal adipose tissue

Treatment with *1,25D*_3 _to non-diabetic rats increased both the protein content and the indicator of cell size (protein/DNA) in the kidney, liver and adipose tissue of these animals while the DNA and RNA content remained unaltered (Table 2). Streptozotocin injection incremented the protein content in these three tissues, accompanied in the case of kidney with an increment of the protein/DNA ratio and in the case of the liver with an increment of the DNA content. Treatment with *1,25D*_3 _of streptozotocin-induced diabetic rats practically did not modify neither the high values of protein induced by the diabetes in the three tissues nor the increases in the DNA content in kidney and liver. In addition, this treatment did not alter the RNA content in any of these three tissues (Table 2).

**Table 2:** Total DNA, RNA and protein content in different tissues from the four groups of rats

	***Control***	***1,25D***_3_	***STZ***	***STZ + 1,25D***_3_
**Kidney**				
**DNA (mg/g)**	3.0 ± 0.1	2.8 ± 0.2	3.3 ± 0.5	3.4 ± 0.1^ab^
**Protein(mg/g)**	69 ± 2	120 ± 5^a^	134 ± 33^a^	153 ± 29^ab^
**Prot/DNA (mg/mg)**	18 ± 5	43 ± 2^a^	41 ± 10^a^	45 ± 10^a^
**RNA (mg/g)**	1.3 ± 0.1	1.6 ± 0.2	1.3 ± 0.3	1.7 ± 0.3
**Liver**				
**DNA (mg/g)**	1.9 ± 0.2	1.5 ± 0.2	2.9 ± 0.6^ab^	2.4 ± 0.2^ab^
**Protein(mg/g)**	105 ± 19	146 ± 17^a^	203 ± 27^ab^	182 ± 15^a^
**Prot/DNA (mg/mg)**	52 ± 9	99 ± 11^a^	67 ± 5	76 ± 3^a^
**RNA (mg/g)**	3.9 ± 0.3	3.2 ± 0.4	3.5 ± 0.4	3.8 ± 0.2
**Adipose tissue**				
**DNA (mg/g)**	0.2 ± 0.06	0.2 ± 0.02	0.3 ± 0.03	0.3 ± 0.04
**Protein(mg/g)**	5.1 ± 1	8.2 ± 0.8^a^	7.9 ± 0.5^a^	6.1 ± 1
**Prot/DNA (mg/mg)**	24 ± 5	47 ± 6^a^	25 ± 1^b^	20 ± 3
**RNA (mg/g)**	0.1 ± 0.02	0.1 ± 0.01	0.1 ± 0.03	0.1 ± 0.04

Sham-treated rats (*Control*), rats treated with *1,25D*_3 _[150 IU/Kg (3.75 μg/Kg) one a day, for 15 days] (*1,25D*_3_), streptozotocin-induced diabetic rats (*STZ*) and streptozotocin-induced diabetic rats treated with *1,25D*_3 _(*STZ+1,25D*_3_).

Values are the mean ± SEM of 4–6 determinations in each group.

^a ^p < 0.05 vs. *Control*-rats; ^b ^p < 0.05 vs. *1,25D*_3_-rats; ^c ^p < 0.05 vs. *STZ*-rats.

### Insulin receptor mRNA levels in the kidney, liver and epididymal adipose tissue

**Figure 1:**
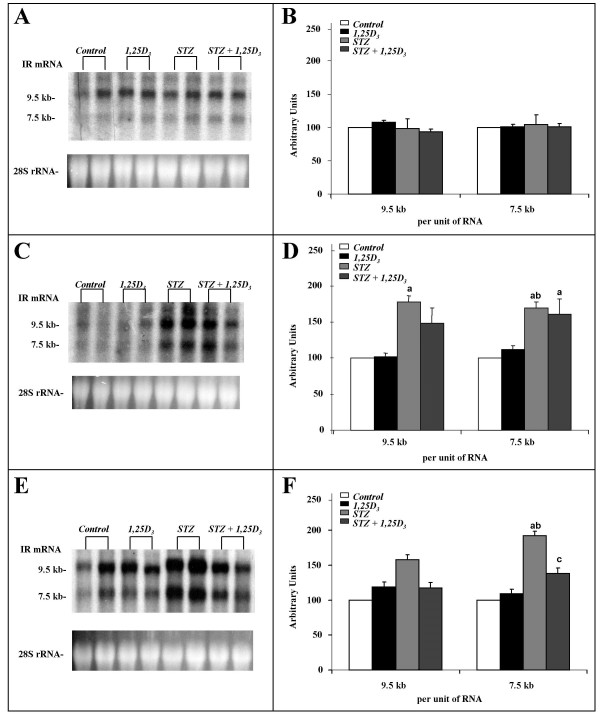
**Renal, hepatic and adipose IR mRNA levels**. Insulin receptor (IR) mRNA levels in the kidney (A, B), liver (C, D) and epididymal adipose tissue (E, F) of sham-treated rats (*Control*), rats treated with *1,25D*_3 _[150 IU/Kg (3.75 μg/Kg) one a day, for 15 days](*1,25D*_3_), streptozotocin-induced diabetic rats (*STZ*) and streptozotocin-induced diabetic rats treated with *1,25D*_3 _(*STZ*+*1,25D*_3_). On the right, the autoradiograph of a representative Northern blot experiment using the kidney (B), liver (D) and adipose tissue (F) of two rats. The sizes of the two major IR mRNA species are shown in the margin. On the left, densitometric analysis of three independent Northern blot experiments on kidney (A), liver (C), and adipose tissue (E). IR mRNA species were quantified separately, normalized to the respective 28S rRNA values and expressed per unit of RNA as a percentage of the values obtained in *Control*-rats (mean ± SEM). ^a ^p < 0.05 vs. *Control*-rats; ^b ^p < 0.05 vs. *1,25D*_3_-rats; and ^c ^p < 0.05 vs. *STZ*-rats.

Northern blot assays of kidney (Figure 1, panels A and B), liver (Figure 1, panels C and D) and epididymal adipose tissue (Figure 1, panels E and F) from control animals revealed two major IR mRNA species of approximately 9.5 and 7.5 Kb in size. The relative amounts of the two IR mRNA species measured as 9.5 Kb/7.5 Kb ratio were: 2.2 ± 0.2 in kidney, 1.2 ± 0.2 in liver and 1.1 ± 0.03 in adipose tissue. Treatment with *1,25D*_3 _to non-diabetic rats did not affect any of the two IR mRNA species in any of the three tissues studied (Figure 1, panels A to F). IR mRNA species were expressed per unit of RNA in view of the observation (Table 2) that treatment with *1,25D*_3 _to both non-diabetic and diabetic rats did not alter RNA content per gram of tissue in any of the three tissues. The induction of diabetes by streptozotocin produced important increments in the levels of both IR mRNA species in liver and adipose tissue but not in kidney. In particular, 78% increase corresponding to the 9.5 Kb and 69% to the 7.5 Kb species in liver (Figure 1, panel D), and 58% increase corresponding to the 9.5 Kb and 92% increase to the 7.5 Kb species in the adipose tissue (Figure 1, panel F). Treatment with *1,25D*_3 _to diabetic rats partially prevented the over-expression of the IR mRNA in the liver (Figure 1, panels C and D) and adipose tissue (Figure 1, panels E and F) of these animals. The 9.5 Kb species was particularly decreased in liver and the 7.5 kb species in the adipose tissue. However, treatment with *1,25D*_3 _to diabetic rats did not affect IR expression in kidney (Figure 1, panels A and B).

### Insulin binding in isolated adipocytes

**Figure 2:**
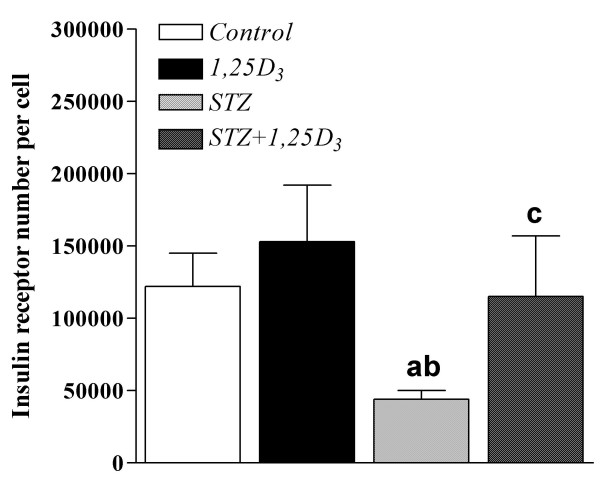
**Insulin receptor number per cell**. Insulin receptor number in epididymal adipocytes from sham-treated rats (*Control*), rats treated with *1,25D*_3 _[150 IU/Kg (3.75 μg/Kg) one a day, for 15 days] (*1,25D*_3_), streptozotocin-induced diabetic rats (*STZ*) and streptozotocin-induced diabetic rats treated with *1,25D*_3 _(*STZ*+*1,25D*_3_). Values are the mean ± SEM of 3–9 determinations in each group. ^a ^p < 0.05 vs. *Control*-rats; ^b ^p < 0.05 vs. *1,25D*_3_-rats; and ^c ^p < 0.05 vs. *STZ*-rats.

Insulin binding studies showed that treatment with *1,25D*_3 _to non-diabetic rats did not alter IR number or IR affinity as reflected by the ED:50 value in adipocytes of these animals (Figure 2 and Table 3). Streptozotocin injection produced a 64% decrease in the number of IRs without altering IR affinity. Treatment with *1,25D*_3 _to streptozotocin-induced diabetic rats inverted the decrease in the IR number induced by the diabetes to values not significantly different from those observed in control adipocytes and without altering IR affinity (Figure 2 and Table 3).

**Table 3:** Insulin binding parameters in epididymal adipocytes from the four groups of rats

	***Control***	***1,25D***_3_	***STZ***	***STZ + 1,25D***_3_
**Insulin receptor number**	122000 ± 23000	153000 ± 39000	44000 ± 6000^ab^	115000 ± 42000^c^
**ED:50 value**	3.0 ± 0.4	4.2 ± 1.4	3.7 ± 1.4	4.8 ± 0.9
**Adipose tissue weight (g)**	3.2 ± 0.2	3.0 ± 0.3	1.6 ± 0.2^ab^	2.0 ± 0.2^a^
**Adipocyte diameter (μm)**	50 ± 1	51 ± 1	39 ± 3^ab^	40 ± 1^ab^
**Insulin receptor number/μm**^2^	16 ± 3	19 ± 5	9 ± 1^ab^	23 ± 8

Sham-treated rats (*Control*), rats treated with *1,25D*_3 _[150 IU/Kg (3.75 μg/Kg) one a day, for 15 days] (*1,25D*_3_), streptozotocin-induced diabetic rats (*STZ*) and streptozotocin-induced diabetic rats treated with *1,25D*_3 _(*STZ+1,25D*_3_).

Values are the mean ± SEM of 3–9 determinations in each group.

^a ^p < 0.05 vs. *Control*-rats; ^b ^p < 0.05 vs. *1,25D*_3_-rats; ^c ^p < 0.05 vs. *STZ*-rats.

Given that both the weight of the adipose tissue and the diameter of the adipocytes were markedly decreased by the diabetes (Table 3), we also calculated the IR number per cell surface area (μm^2^). In these conditions, although the effect of the diabetes decreasing IR number was somewhat minor (44%), the tendency of *1,25D*_3 _to normalize the IR number was also evident (Table 3).

### Glucose transport in isolated adipocytes

**Figure 3:**
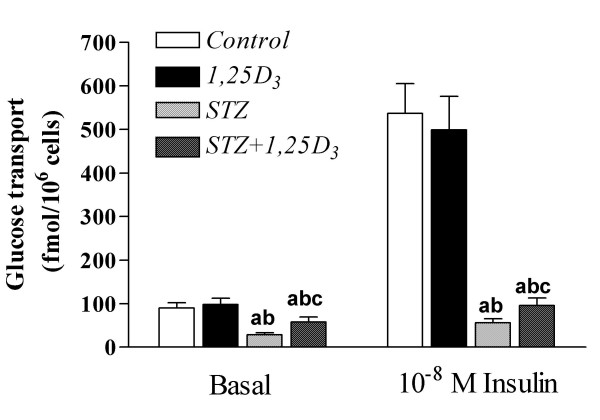
**Basal and insulin stimulated glucose transport**. Basal and insulin stimulated glucose transport in epididymal adipocytes from sham-treated rats (*Control*), rats treated with *1,25D*_3 _[150 IU/Kg (3.75 μg/Kg) one a day, for 15 days] (*1,25D*_3_), streptozotocin-induced diabetic rats (*STZ*) and streptozotocin-induced diabetic rats treated with *1,25D*_3 _(*STZ*+*1,25D*_3_). Values are the mean ± SEM of 6–7 determinations within each group. ^a ^p < 0.05 vs. *Control*-rats; ^b ^p < 0.05 vs. *1,25D*_3_-rats; and ^c ^p < 0.05 vs. *STZ*-rats.

Treatment with *1,25D*_3 _to non-diabetic rats did not alter basal or insulin-stimulated glucose transport in adipocytes from these animals (Figure 3). The injection of streptozotocin clearly decreased both basal and insulin-stimulated glucose transport. The treatment with 1,25D_3 _to streptozotocin-induced diabetic rats, improved by 107% the decreased basal glucose transport and by 71% the insulin-stimulated glucose transport in adipocytes from these diabetic animals (Figure 3).

### Computer analysis of DNA sequences in the rat IR gene promoter

**Figure 4:**
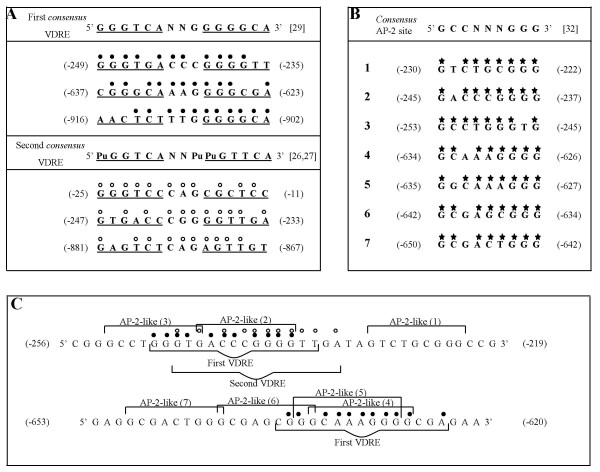
**Panel A: Computer search of vitamin D response elements (VDREs) in the rat insulin receptor (IR) gene promoter**. Potential VDRE sequences in the rat IR gene promoter were identified by their homology with two *consensus *VDRE sequences using the SEQFIND programme developed in our laboratory. Nucleotides identical to the first VDRE *consensus *are labelled by filled circles (●) and nucleotides identical to the second VDRE *consensus *are labelled by empty circles (○). Panel B: Computer search of AP-2-like sites in the rat insulin receptor (IR) gene promoter. Potential AP-2-like sites in the rat IR gene promoter were identified by their homology with the AP-2 *consensus *(5'GCCN3GGG3') [32] using the SEQFIND programme. We detected 17 AP-2-like sites showing a difference of only 1 base from this *consensus*. Of these, only 7 AP-2-like sites flanking or overlapping the potential VDRE sequences in panel A are shown. Nucleotides identical to the AP-2 *consensus *are labelled by stars (⋆). Panel C: Candidate VDRE sequences overlapped by AP-2-like sites in the rat insulin receptor gene promoter. Nucleotides identical to the first VDRE *consensus *are labelled by filled circles (●) and nucleotides identical to the second VDRE *consensus *are labelled by empty circles (○)

We performed a computer search of virtual VDRE sequences in the rat IR gene promoter by homology with two *consensus *VDRE sequences. The first *consensus *(5'**GGGTCA**NNG**GGGGCA**3') was previously compiled and reported by us from a series of described functional VDRE sequences in various 1,25D_3_-stimulated promoters [29]. The search with this first *consensus *indicated no sequence identical to this VDRE sequence, and neither was any sequence found to show 1, 2, or 3 base variations. Nevertheless, we detected three virtual VDRE sequences showing a difference of four bases from this *consensus*: -249/-235, -637/-623 and -916/-902 (Figure 4, panel A). Comparison of the 3' half-element of each of these sequences with the 3' half-element of this first VDRE *consensus*, indicated complete homology only in the case of the -916/-902 sequence, and two base variations in the other two (Figure 4, panel A).

The second computer search of virtual VDREs, now by homology with the second *consensus *VDRE sequence (5'**PuGGTCA**NNPu**PuGTTCA**3'), was also performed. Our *consensus *and the latter proposed by Colnot *et al. *[27], and Haussler *et al. *[26] have identical 5' half-elements, but ours contains two GG instead of two TT, in the 3' half-element. The spacers are identical, with a G or Pu at position 3, which appears to be important in VDR binding. The results of this computer search by homology with this second *consensus *indicated no sequence identical to this VDRE, and neither was any sequence found to show 1, 2, or 3 base variations. Nevertheless, we detected three virtual VDRE sequences showing a difference of four bases from this second *consensus*: -25/-11, -247/-233 and -881/-867 (Figure 4, panel A). Of these, the -247/-233 sequence was in part the same as that of the -249/-235 sequence detected with the first *consensus *(Figure 4, panel A). However, the 3' half-element of this second sequence has only one base variation instead of two that the first sequence had (Figure 4, panel A). Therefore, in principle there were six virtual VDRE sequences (Figure 4, panel A).

The next step was to check for the possibility of cis-elements related to these virtual VDRE sequences in the rat IR gene promoter. Thus, a computer search of AP-1 and AP-2 sites by homology with the AP-1 site consensus (5'TGAC/GTCA3') [31], and the AP-2 site consensus (5'GCCN3GGG3') [32], respectively, was performed.

Exploring the AP-1 sites, we detected two AP-1-like sites showing a difference of only one base from this consensus: the -374/-368 (5'TGTCTCA3') and the -963/-957 (5'TGAGCCA3'). However, these two sites are neither adjacent nor overlapped in any of our virtual VDRE sequences. Nevertheless, as far as we know, this is the first mention of these AP-1-like sites in the rat IR gene promoter. With respect to the AP-2 sites, we detected seventeen AP-2-like sites showing a difference of only one base from this *consensus*: -71/-63, -79/-71, -171/-163, -195/-187, -230/-222, -245/-237, -253/-245, -405/-397, -465/-457, -480/-472, -545/-537, -634/-626, -635/-627, -642/-634, -650/-642, -1087/-1079, -1088/-1080. Of these AP-2-like sites, only seven are included in panel B of the Figure 4. These were those flanking or overlapping some of the potential VDRE sequences indicated in panel A of the Figure 4. Of them, the -230/-222 (1) was located downstream, and the -245/-237 (2) and the -253/-245 (3) were overlapping the virtual VDRE sequences: -249/-235 and -247/-233, as shown in Figure 4, panel C. Moreover, the AP-2-like sites: -634/-626 (4) and -635/-627 (5) (Figure 4, panel B) were overlapping the virtual VDRE sequence: -637/-623 (Figure 4, panel C), and the AP-2-like sites: -642/-634 (6) and -650/-642 (7) (Figure 4, panel B) were located *in tandem *upstream of this last virtual VDRE sequence: -637/-623 (Figure 4, panel C).

Therefore, we postulate as candidate VDRE sequences overlapped by AP-2-like sites those represented in Figure 4, panel C. One, located between -256 and -219 bp with three AP-2-like sites, and the other extending from -653 and -620 bp of the rat IR gene promoter with four AP-2-like sites. Separately or together, these VDRE sequences could form a locus that may respond to 1,25D3 via activation of VDR.

## Discussion

### Effects of treatment with *1,25D*_3 _to non-diabetic rats

Treatment with *1,25D*_3 _to non-diabetic rats increased body weights but it did not affect any parameter measured in the plasma or urine of these animals, including the plasma levels of calcium, phosphorus and 25-hydroxyvitamin D_3_. Indeed, we did not detect significant differences in calcium and phosphorus, despite the increases of approximately 30%. In relation to 25-hydroxyvitamin D_3 _a number of studies [33] has shown that human serum values of 25-hydroxyvitamin D_3 _were maintained within a normal range across vitamin D supplies from 80 to 1000 IU daily, indicating the existence of an important homeostatic control system regulating human 25(OH)D_3 _serum concentration. Considering this, in the present study we hypothesize the existence of a similar counter regulatory mechanism in the rat. This mechanism could maintain rat plasma values of 25-hydroxyvitamin D_3 _within the observed normal range of 15–19 ng/ml, with vitamin D supplies of 150 IU daily administered by injection plus the vitamin D consumed in the diet.

Treatment with *1,25D*_3 _increased the protein content and the indicator of cell size (protein/DNA) in the kidney, liver, and adipose tissue of non-diabetic rats, although the DNA and RNA content remained unaltered. These increments might indicate hypertrophy. However, although treatment with *1,25D*_3 _increased rat body weights, it did not particularly augment the weight of these tissues. Another explanation is that *1,25D*_3 _might cause hyperplasia. Regarding this, conflicting results have been reported [34,35].

We also observed that treatment with *1,25D*_3 _to non-diabetic rats did not affect the expression of any of the two 9.5 and 7.5 Kb IR mRNA species present in kidney, liver, and adipose tissue. Our group and others have previously reported the presence of these species and their relative proportions in rat tissues [21,36]. Since there is only one rat IR gene, variation in transcript length may reflect alternative RNA processing events, such as polyadenylation at different 3' end sites in the final untranslated exon [37].

In addition, we detected that treatment with *1,25D*_3 _to non-diabetic rats affected neither the IR number nor the insulin response in terms of glucose transport in isolated adipocytes from these animals.

The lack of effects of *1,25D*_3 _on IR expression and insulin activity in tissues of non-diabetic rats could be related to the absence of VDR in these tissues. However, both the VDR protein and VDR mRNA have been detected in the rat kidney [38,39] and liver [40,41], and while a VDR-like protein was identified in 3T3-L1 adipose cells [42] VDR mRNA has been found in rat perirenal adipose tissue and 3T3-L1 adipose cells [43]. Another possibility could be the absence of regulation of VDR by its ligand in these tissues. Such lack of regulation has been described in the kidney [39]. In the liver there are no previous data, while in adipose cells treatment with *1,25D*_3 _led to an increase in VDR mRNA levels [43] and a decrease in VDR protein [35]. Regarding this and given our previous experience in determining both VDR protein and VDR mRNA expression and their regulation by *1,25D*_3 _in human cells [6,7], we determined the expression of this receptor and its regulation by 1,25D_3 _at the protein level in the kidney, liver and adipose tissue of non-diabetic rats. Our results showed a VDR protein only in the kidney (data not shown). More experiments are necessary to confirm this.

### Effects of treatment with *1,25D*_3 _to streptozotocin-induced diabetic rats

Treatment with *1,25D*_3 _to streptozotocin-induced diabetic rats increased their body weight, but it did not revert the hyperglycemia and the hypoinsulinemia provoked by the streptozotocin. We also detected normal levels of 25-hydroxyvitamin D_3 _in streptozotocin-induced diabetic rats in accordance with other authors [30]. These unchanged levels of 25-hydroxyvitamin D_3 _in streptozotocin-induced diabetic rats neither were altered by the treatment with *1,25D*_3_, possibly due to the postulated plasma homeostatic control system that could regulate 25-hydroxyvitamin D_3 _plasma levels also in the absence of insulin in both groups of diabetic rats. In addition, we observed increased plasma levels of calcium and phosphorus after the treatment with *1,25D*_3_. With regard to these last parameters, it is known that calcium *per se *is important for insulin secretion, as well as for correction of glucose intolerance [44,45]. Moreover, calcium and phosphorus have been described as regulators of VDR in renal and intestinal tissues [39,46].

Treatment with *1,25D*_3 _to streptozotocin-diabetic rats did not modify the glycosuria and the ketonuria induced by the diabetes, but partially decreased the number of leukocytes in urine. This latter effect could be due to the broadly reported anti-inflammatory effects of *1,25D*_3 _and/or to a possible anti-proliferative effect of this hormone, as observed by our group and others in cell culture [6,33].

With respect to tissue effects of *1,25D*_3 _in streptozotocin-induced diabetic rats, we observed the permanence of high protein and DNA content values induced by the diabetes, accompanied by slight increases of the protein/DNA ratio values in the kidney and liver, but not in the adipose tissue. This suggests a certain hypertrophy produced by the action of *1,25D*_3 _in these two tissues. However, although treatment with *1,25D*_3 _to streptozotocin-diabetic rats increased rat body weights, it did not increase the weight of the kidney, liver or adipose tissue. The other explanation, hyperplasia, has conflicting results [33,34].

The induction of diabetes by streptozotocin did not affect IR mRNA species in the kidney. This finding was not in accord with previous results of increased renal IR mRNA levels, quantified by slot blot, in a similar rat model [22]. This may reflect the failure to alter VDR number in the kidney of streptozotocin-diabetic rats [34]. The treatment with 1,25D_3 _to streptozotocin-induced diabetic rats neither affected IR mRNA species in the kidney.

Contrarily, streptozotocin produced an important increment in the levels of both IR mRNA species in liver. These findings agree with previous results of increased IR mRNA levels quantified by slot blot [22], Northern blot analysis [21,47] and increased IR gene transcription [21] in the liver of streptozotocin-induced diabetic rats. Streptozotocin also increased the levels of both IR mRNA species in the adipose tissue. This increment represents the first demonstration of an *in vivo *regulation of IR mRNA levels in the adipose tissue of streptozotocin-induced diabetic rats.

In addition, we observed that the treatment with *1,25D*_3 _to streptozotocin-induced diabetic rats partially prevented the over-expression of IR mRNA induced by the diabetes in the liver and adipose tissue of these animals. The 9.5 Kb IR mRNA species was particularly decreased by *1,25D*_3 _in the liver, and the 7.5 Kb species in the adipose tissue. Therefore, these results provide the first demonstration of an *in vivo *tissue-specific regulation of rat IR mRNA levels by *1,25D*_3 _in streptozotocin-induced diabetic rats.

A role for VDR in such genomic actions of *1,25D*_3 _in liver and adipose tissue is possible, although to our knowledge the regulation of VDR by *1,25D*_3 _has not yet been studied in these tissues of streptozotocin-diabetic rats.

The correction by *1,25D*_3 _of the over-expression of IR expression at the RNA level in the liver and adipose tissue of streptozotocin-diabetic animals could represent a beneficial fact of amelioration of the diabetes. In this sense, other facts of amelioration of diabetes were the almost normalization of the number of IRs in adipocytes of streptozotocin-diabetic animals treated with *1,25D*_3_, without alterations in receptor affinity, and the improvement of both basal and insulin-stimulated glucose transport in adipocytes of these diabetic animals treated with *1,25D*_3_.

The present *in vivo *results do not allow to determine whether the prevention by *1,25D*_3 _of the over-expression of IR mRNA induced by the diabetes in liver and adipose tissue of streptozotocin-diabetic animals is due to transcriptional and/or post-transcriptional regulation. Nevertheless, our previous *in vitro *results demonstrated that *1,25D*_3 _increased IR mRNA levels via mechanisms involving direct transcriptional activation of the human IR gene [5-7]. Moreover, we identified an active VDRE in the human IR gene promoter that accounted for the transcriptional induction of this gene by *1,25D*_3 _in U-937 cells [29].

Therefore, in support of the possible participation of a transcriptional regulation by *1,25D*_3 _in the present in vivo processes, we have detected by computer search in the rat IR gene promoter two candidate VDREs overlapped by various AP-2-like sites. One VDRE is located between -256 and -219 bp, while the other extends from -653 and -620 bp of the rat IR gene promoter (Figure 4, panel C). Individually or in conjunction, these potential VDREs could form a locus that may respond to *1,25D*_3 _via activation of VDR. This locus may mediate the cross-talk between vitamin D and insulin signalling, although this remains to be determined.

## Conclusion

The present results indicate that while treatment with *1,25D*_3 _had practically no effect on non-diabetic rats, the same treatment in streptozotocin-induced diabetic rats corrected in part the over-expression of the IR gene in liver and adipose tissue, although it did not revert the hyperglycemia, hypoinsulinemia, glycosuria or ketonemia of these diabetic animals. At the same time, it produced normalization of the IR number without alterations in the receptor affinity, but with an improvement of the insulin response in terms of glucose transport in adipocytes of these diabetic animals. These genomic actions of *1,25D*_3 _could represent beneficial facts of amelioration of diabetes via mechanisms, possibly involving direct transcriptional activation of the rat IR gene. A computer search in the rat IR promoter revealed the existence of two candidate vitamin D response element (VDRE) sequences, located at -256/-219 bp and -653/-620 bp and overlapped by three and four AP-2-like sites respectively. These candidate VDREs may respond to *1,25D*_3 _via activation of the vitamin D receptor, although this remains to be investigated.

## Methods

### Animals and sampling

Four groups of male Wistar rats weighing 200–220 g at the onset were used in this study. They were kept under standard conditions of light and temperature and given free access to tap water and standard laboratory chow (Panlab, A04) containing 8.8 mg/g of calcium, 5.9 mg/g of phosphorus and 1.5 IU/g of vitamin D. The first group comprised of non-diabetic rats receiving sham-treatments during the 15 days of the experimental period (*Control-rats*). The second group included non-diabetic rats i.*p*. injected with 1,25D_3 _(Calcijex, Abbot) [150 IU/Kg (3.75 μg/Kg) one a day, for 15 days] (*1,25D*_3_-*rats*). The third group consisted of rats rendered diabetic by a single injection of streptozotocin (Sigma) [65 mg/kg] on the 8th day of the 15-day period (*STZ-rats*). Finally, the fourth group comprised of rats rendered diabetic by streptozotocin on the 8th day, and i.p. injected with *1,25D*_3 _[150 IU/Kg (3.75 μg/Kg) one a day, for 15 days] (*STZ*+*1,25D*_3_-*rats*). The National Guide for the Care and Use of Laboratory Animals was strictly followed during this study.

Urine samples were collected before decapitation without anaesthesia and trunk blood samples were recovered for plasma measurements. On sacrifice, the kidney, liver and epididymal adipose tissue were immediately removed, individually packed and frozen in liquid nitrogen for nucleic acid and protein determinations. Isolated adipocytes were obtained from fresh epididymal adipose tissue for insulin binding and insulin activity assays.

### Analytical methods

Different parameters in urine were measured using the Combur Test (Roche). Plasma insulin levels were determined by radioimmunoassay using rat insulin as standard. Plasma concentrations of glucose, 25-hydroxyvitamin D_3_, calcium, phosphorus and proteins were estimated using commercially available techniques. The total DNA and RNA contents of the homogenates from individual tissues were estimated by spectrofluorometry. The protein content was determined by the Bradford method.

### RNA blot assays

For Northern blot assays, RNA samples (40 μg) of kidney, liver and epididymal adipose tissue, were denatured, electrophoresed in 1.1% agarose-formaldehyde gels and blotted onto nylon membranes [36]. Ethidium bromide staining of the 28S and 18S ribosomal RNAs was routinely checked before blotting as a control of sample loading and after blotting as a control of RNA transfer. RNA blots were prehybridized, hybridized with excess [^32^P]-labelled probe (the 0.98-Kb rat IR specific EcoR1 fragment of the p16 clone, gift from Prof. Goldstein), washed under stringent conditions and finally autoradiographed, as described previously [36]. The autoradiographs were scanned with a laser densitometer and the readings normalized with the respective amounts of 28S rRNA, as revealed by ethidium bromide.

### Insulin binding assays

Binding assays were carried out as described previously [48]. In brief, isolated adipocytes (0.25 × 10^6 ^cells/ml) were incubated at 30°C for 30 min in 400 μl Krebs-Hepes buffer pH 7.6 with mono-[^125^I]-insulin (0.2 × 10^-9 ^M) (NEN Life Sciences), in the presence or absence of increasing concentrations (0.2 × 10^-10 ^to 0.5 × 10^-7 ^M) of unlabelled insulin (Sigma). Non-specific binding was determined in the presence of 1 × 10^-7 ^M unlabelled insulin and subtracted from each point. The adipocyte number was determined with a Neubauer-type hemocytometer and cell viability (assessed by 0.2% trypan blue exclusion) was greater than 90%. Measurements of the cell diameter were also performed. The data were analysed by the Scatchard method using the LIGAND program of Munson and Rodbard [49].

### Glucose transport determinations

Glucose transport was measured in isolated adipocytes by determining the uptake of D-[^14^C(U)]-glucose (NEN Life Sciences) at trace concentrations using our modification of the method of Kashiwagi et al. [50]. This method is based on the premise that glucose uptake provides a measure of glucose transport when studies are carried out at very low glucose concentrations. In brief, 0.25 × 10^6 ^adipocytes/ml were incubated with 5 × 10^-7 ^M D-[^14^C(U)]-glucose for 1 h at 37°C in the presence and absence of a concentration of insulin (10^-8 ^M) that gave maximal glucose transport in earlier experiments [6]. Assays were stopped by washing the cells with ice-cold PBS. The cells were solubilized in 0.5% SDS and 0.1 N NaOH and the associated radioactivity was counted.

### Computer analysis of DNA sequences in the rat IR gene promoter

The identification of virtual VDRE sequences in the *Rattus norvegicus *partial IR promoter [GenBank:AJ006071] was carried out by homology with two *consensus *VDRE sequences using the SEQFIND programme developed in our laboratory and previously employed in the computer analysis of various DNA sequences including VDREs [29]. The first *consensus *(5'**GGGTCA**NNG**GGGGCA**3') had been compiled and reported by us from a series of described functional VDRE sequences in various 1,25D_3_-stimulated promoters [29]. The second *consensus *VDRE sequence (5'**PuGGTCA**NNPu**PuGTTCA**3') had been proposed by Colnot *et al*. [27], and Haussler *et al*. [26]. The presence of AP-1 and AP-2 sites flanking or overlapping the potential VDREs were identified by their homology with the AP-1 site *consensus *(5'TGAC/GTCA3') [31] and the AP-2 site *consensus *(5'GCCN3GGG3') [32] respectively, also using the SEQFIND programme.

### Statistical analysis

Unless otherwise indicated, the data are expressed as the mean ± SEM. A comparison between the groups was carried out using the two-tailed, unpaired Student *t*-test and/or ANOVA comparison, as appropriate. Differences were considered statistically significant when p < 0.05.

## List of abbreviations

1,25D_3_: 1,25-dihydroxyvitamin D_3_; IR: insulin receptor; VDR: vitamin D receptor; RXR: retinoid × receptor; VDRE: vitamin D response element; STZ: streptozotocin.

## Authors' contributions

CC conceived the concept and design of the study, carried out the binding and activity studies, analyzed the computer data of DNA sequences and provided drafting of the article. BM carried out the rat models and the determinations in plasma and urine of these animals and performed the RNA blot assays. She also supplied statistical expertise, collected and assembled the data. MG-A participated in the identification by computer analysis of potential VDRE sequences and provided computer support. All authors read and approved the final manuscript.
